# Measuring Molecular Diffusion in Dynamic Subcellular Nanostructures by Fast Raster Image Correlation Spectroscopy and 3D Orbital Tracking

**DOI:** 10.3390/ijms23147623

**Published:** 2022-07-10

**Authors:** Filippo Begarani, Francesca D’Autilia, Gianmarco Ferri, Luca Pesce, Fabio Azzarello, Valentina De Lorenzi, William Durso, Ambra Del Grosso, Marco Cecchini, Francesco Cardarelli

**Affiliations:** 1 NEST-Scuola Normale Superiore, 56127 Pisa, Italy; filippo.begarani@sns.it (F.B.); gianmarco.ferri@sns.it (G.F.); luca.pesce1@sns.it (L.P.); fabio.azzarello@sns.it (F.A.); valentina.delorenzi@sns.it (V.D.L.); william.durso@sns.it (W.D.); marco.cecchini@nano.cnr.it (M.C.); 2 Center for Nanotechnology Innovation@NEST (CNI@NEST), 56127 Pisa, Italy; francesca.dautilia@iit.it; 3 NEST, Istituto Nanoscienze-CNR, Piazza S. Silvestro, 12, 56127 Pisa, Italy; ambra.delgrosso@sns.it

**Keywords:** diffusion, correlation spectroscopy, fluorescence, living cells, subcellular scale, nanoscale, lysosome, insulin secretory granule

## Abstract

Here we provide demonstration that fast fluorescence fluctuation spectroscopy is a fast and robust approach to extract information on the dynamics of molecules enclosed within subcellular nanostructures (e.g., organelles or vesicles) which are also moving in the complex cellular environment. In more detail, Raster Image Correlation Spectroscopy (RICS) performed at fast timescales (i.e., microseconds) reveals the fast motion of fluorescently labeled molecules within two exemplary dynamic subcellular nanostructures of biomedical interest, the lysosome and the insulin secretory granule (ISG). The measurement of molecular diffusion is then used to extract information on the average properties of subcellular nanostructures, such as macromolecular crowding or molecular aggregation. Concerning the lysosome, fast RICS on a fluorescent tracer allowed us to quantitatively assess the increase in organelle viscosity in the pathological condition of Krabbe disease. In the case of ISGs, fast RICS on two ISG-specific secreting peptides unveiled their differential aggregation propensity depending on intragranular concentration. Finally, a combination of fast RICS and feedback-based 3D orbital tracking was used to subtract the slow movement of subcellular nanostructures from the fast diffusion of molecules contained within them and independently validate the results. Results presented here not only demonstrate the acquired ability to address the dynamic behavior of molecules in moving, nanoscopic reference systems, but prove the relevance of this approach to advance our knowledge on cell function at the subcellular scale.

## 1. Introduction

A major challenge of present (and future) biophysics is to address quantitatively how molecules dynamically fulfill their physio(patho)logical role in living cells. Many quantitative biophysical approaches were pursued in the last few decades to measure crucial molecular parameters (e.g., diffusion, concentration, binding constants, oligomerization state, etc.) directly within living cells. Yet, in many circumstances, molecular behavior is destined to remain elusive. For instance, one of the natural conditions of living matter is that of subcellular structures, such as transport vesicles (e.g., endocytic and secretory vesicles), organelles (e.g., lysosomes, mitochondria, etc.) or even entire cellular protrusions (e.g., dendritic spines). Here, a complex realm of molecular processes are hosted in a tiny reference system (typically 50–500 nm in size, comparable to or below the resolution of standard optical setups) that is also rapidly changing position in the complex 3D intracellular environment. Quantitative probing of molecular processes in such conditions is inevitably challenged by the need to satisfy, simultaneously, three crucial requisites in a single measurement: (*i*) nanometer spatial resolution to address the spatial scale of molecules, (*ii*) micro-to-millisecond temporal resolution to probe the characteristic timescale of molecular processes and (*iii*) large volume sampling to compensate for the 3D evolution of the entire reference system. Concerning point (*i*), a number of experimental methodologies are still available to probe living matter at the nanoscale (even a tiny fraction of visible light wavelength), thus moving the nominal spatial resolution far beyond the diffraction limit. These include, among others, methods based on shaping the excitation light beam, such as STimulated Emission Depletion (STED [1]), ground-state depletion [2], REversible Saturable Optical Linear Fluorescence Transition (RESOLFT [3]) or Structured Illumination Microscopy (SIM [4,5,6]), and methods which combine specific spectral properties of the fluorescent probes (e.g., photoactivation and on/off switching) with single-molecule localization, such as STochastic Optical Reconstruction Microscopy (STORM [7,8]), PhotoActivated Localization Microscopy (PALM [9]) and Point Accumulation for Imaging in Nanoscale Topography (PAINT [10,11]). Unfortunately, regardless of the achievable spatial resolution of the measurement, high temporal resolution (requisite (*ii*)) and large volume sampling (requisite (*iii*)) remain two mutually dependent parameters, i.e., the larger the volume to probe, the lower the temporal resolution achievable. Such limitations are driving new exciting developments in the field. For instance, Schneider and co-workers successfully combined STED-based imaging with electro-optical scanning technologies to obtain the line-scanning frequency of 250 kHz [12]: using single-molecule localization and the 70 nm static resolution provided by STED, the authors addressed the dynamics of fluorescently labeled vesicles in living Drosophila or HIV-1 particles in cells reaching a final temporal resolution of about 5–10 milliseconds. More recently, a straightforward combination of STED and local excitation-intensity zeros for localizing emitters was proposed, pushing 3D localization experiments to unprecedented spatial (~1 nanometer) and temporal (~100 ms) resolutions [13,14,15], even in a standard fluorescence microscope [16]. This method, named MINFLUX, was successfully applied to track the position in time of, among others, single 30S ribosomal subunits in the cytoplasm of *E. coli* [13], fluorescently labeled lipids on model membranes [16] and fluorescently labeled DNA origami in vitro [14]. However, the ability of the techniques discussed so far to measure the dynamic properties of molecules in the particular case in which they are hosted by also dynamic nanostructures still remains to be tested by experiments. Thus, at present, addressing the complex realm of molecular processes ongoing within dynamic subcellular nanostructures remains an unattained task. In this context, some of us highlighted how the performance of virtually any fluorescence-based optical microscopy strategy, irrespective of its nominal spatial resolution, can be enhanced by the use of Fluorescence Correlation Spectroscopy (FCS) analytical tools [17]. In fact, FCS tools endow optical microscopy with single-molecule sensitivity in the presence of many molecules, with no need to dwell on any of them. Also, FCS tools afford a quantitative description of the average dynamic behavior of molecules at a resolution limited only by the achievable acquisition speed [17]. For instance, building on the original concept of Raster Image Correlation Spectroscopy (RICS [18,19]), some of us were able to probe the dynamic behavior of molecules in cells down to the temporal scale of about 1 microsecond, resolving average protein displacements in the cytoplasm of about 20 nanometers [20,21]. Yet, it should be noted that such a successful experiment was conducted in a 3D biological environment (i.e., the cell interior) that is inherently static on the timescale of the molecular processes under study (i.e., protein diffusion). The potential of the method to study molecules enclosed within dynamic subcellular nanosystems was tested so far only in silico [17]. To tackle this issue, here we demonstrate that RICS in tunable timescales is able to extract the diffusivity of a large set of molecules enclosed within dynamic subcellular nanostructures. It is well-known that the diffusion coefficient (*D*) of (spherical) molecules can be related to relevant experimental parameters through the Stokes–Einstein equation:

(1) $$D=\frac{k_{B}T}{6\pi \eta r}$$

where *k_B_* is the Boltzmann’s constant, *T* is the absolute temperature, *ƞ* is the dynamic viscosity and *r* is the radius of the diffusing molecule. Thus, it should be clear that measuring molecular diffusivity in the subcellular compartments defined here would give access to information on at least two parameters of biological interest: the *viscosity* of the compartment and the apparent radius of the measured molecule. Of note, the former is supposed to be finely regulated in virtually any cellular/subcellular compartment according to the ongoing molecular reactions (and de-regulated, eventually, in pathological phenotypes). The latter, however, will increase or decrease according to molecular aggregation or degradation, respectively, thus providing precious information on the identity of the diffusing species in selected experimental conditions. To prove the potency of the proposed approach, we first calibrated the protocol by using standard test fluorophores both in vitro and in living cells. Then we addressed two biological problems in which measuring the diffusion of molecules at the subcellular scale is of biomedical interest. In brief, molecular diffusion was measured in (*i*) trafficking lysosomes in both physiological and pathological (Krabbe’s disease) conditions and (*ii*) trafficking insulin secretory granules (ISGs) for two different protein markers with similar molecular weight (MW), showing their propensity to aggregate in a concentration-dependent fashion. Independent validation of obtained results was achieved by using feedback-based 3D orbital tracking [22] as a direct means to subtract the slow movement of subcellular nanostructures from the fast diffusion of molecules contained.

It is worth mentioning that all experiments were conducted in a standard, diffraction-limited optical setup operating in raster-scan mode, with no need of complex instrumentation, optics or analytical tool/software. We expect broad applications of this approach for studying the dynamic behavior of molecules in the natural condition of living matter at the subcellular scale.

## 2. Results and Discussion

### 2.1. Fast RICS Captures Molecular Diffusion in Dynamic Nanostructures: Validation Tests in Solution

**Figure 1:**
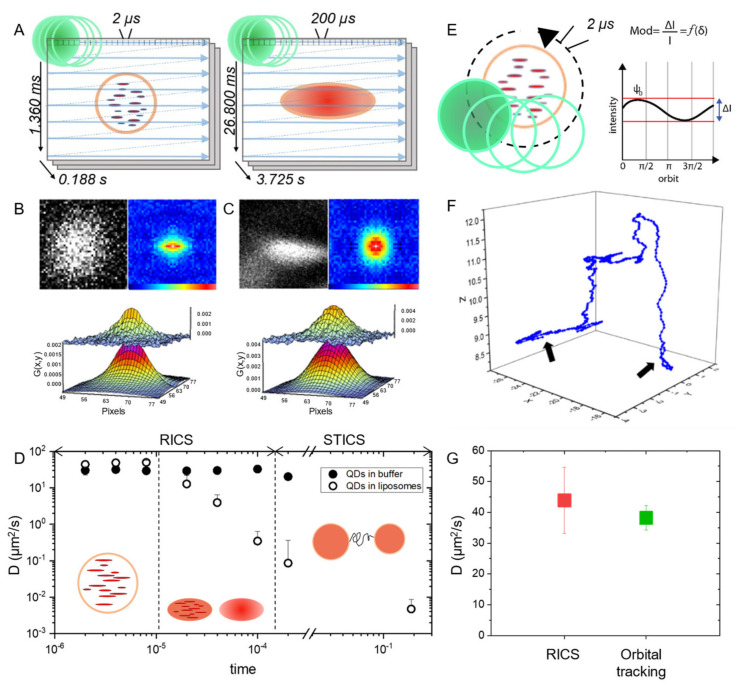
Time-tunable RICS: principle and validation experiments in solution. (**A**) RICS at short pixel dwell times (e.g., 2 µs, which translates into 1360 ms of line-time and 0.188 s of frame-time, left panel) probes the motion of fluorescent molecules embedded within subcellular dynamic nanostructures, as these latter appear immobile during scanning. By contrast, RICS at long pixel dwell times (e.g., 200 µs, that translates into 26.8 ms of line-time and 3.725 s of frame-time) cannot distinguish fast molecular movements within the subcellular nanostructures from the slow movement of the entire nanostructure; this latter component can be isolated and quantified by using STICS. (**B**) Exemplary frame collected at 2 µs pixel dwell time showing a single QD-loaded liposome captured by imaging in solution (upper panel) and the corresponding RICS function (bottom panel), together with fit. (**C**) Exemplary frame collected at 200 µs pixel dwell time showing a single QD-loaded liposome captured by imaging in solution (upper panel) and the corresponding RICS function (bottom panel), together with fit. Note how the RICS function corresponding to 200 µs pixel dwell time is broadened in the y-direction as compared with its 2 µs counterpart. (**D**) Plot of the diffusion coefficients measured at different scan speeds for both QD-loaded liposomes (full dots) and QDs in buffer (empty dots). As schematically represented, at short pixel dwell times (i.e., few microseconds) RICS can probe the movement of the molecules inside the liposome. By contrast, at long pixel dwell times (i.e., hundreds of microseconds), RICS cannot distinguish fast molecular movements within the liposome from the slow movement of the entire liposome; the latter component can be isolated and quantified by STICS. Standard deviations associated to data points are within 10–20% of the mean value: they are not discernible due to the logarithmic scale used on the *y*-axis (**E**) Control experiment using the feedback-based 3D orbital tracking routine. (**F**) Example of trajectory of a QD-loaded liposome in agarose gel obtained using 3D orbital tracking. The average diffusion coefficient of liposomes slowed down in agarose gel is 0.085 ± 0.047 µm^2^/s (expressed as mean ± SD). Ticks on axes represent pixels, thus 50 nm of space distance. (**G**) Plot of the diffusion coefficients (expressed as mean ± SD) of both QDs dispersed in Borate buffer (red, measured by standard RICS at 2 µs/pixel) and QDs loaded into liposomes measured using the feedback-based 3D orbital tracking routine at 4 µs pixel dwell time (green).

The probing of quantitatively dynamic molecular processes in subcellular nanostructures is technically challenged by the continuous 3D movement of the entire reference system. To tackle this issue, consecutive RICS measurements are performed at different scan speeds; in more detail, temporal stacks of confocal images are acquired at progressively increasing pixel dwell times, namely at 2, 4, 8, 20, 40, 100 and 200 µs (the two extreme scan speeds are schematically represented in Figure 1A). Thus, the temporal scale of the experiment is segmented in such a way that the physical origin of the signal fluctuations detected while scanning the single nanostructures of interest varies according to scan speed. In particular, at high scan speeds, the single nanostructures of interest are almost immobile while being imaged and only fast signal fluctuations due to short-range displacements of molecules within the nanostructures will be detected (Figure 1A, left). Then, by decreasing the scan speed, progressively slower signal fluctuations will be detected, due to the overall motion of the nanostructure (Figure 1A, right). In other words, we expect the RICS function to be negligibly affected by the motion of the nanostructure at the highest scan speeds (i.e., its deformation will reflect molecular motion only), while, by contrast, being progressively affected by the motion of the nanostructure at decreasing scan speeds (i.e., its deformation will reflect both molecular motion and nanostructure motion). These expectations are substantiated by the detailed knowledge acquired on the characteristic intracellular diffusivity of virtually any subcellular nanostructure, including those of interest here, that is always in the order of 10^−3^–10^−2^ µm^2^/s [23,24]. Based on this, it can be estimated that an exemplary single nanostructure of 300 nm diameter is fully raster-scanned in approximately 10 ms if imaging is performed at the highest scan speed (i.e., 10 consecutive lines with a pixel size of about 30 nm). In this condition, the nanostructure will move less than 20 nm while being imaged (i.e., less than 10% of its nominal diameter). In the same time window, by contrast, the molecules contained within the nanostructure will substantially explore the space available (e.g., the lumen or the membrane of the nanostructure depending on the specific localization) according to their characteristic diffusion coefficient. This latter can then be captured by the RICS algorithm, which holds this potentiality over a large dynamic range, up to approximately 400 µm^2^/s (typical of small organic fluorophores in solution, see example in Figure S1). Also, a ‘moving average’ filter [18] can be safely set between consecutive frames to subtract the slow movements of the entire nanostructure from the much faster ones of the molecules within it (see Section 3 for more details). These expectations were first validated by preliminary tests using quantum dots (QDs) as fluorescent molecules, either dissolved in solution or loaded into liposomes (with these latter then poured into an agarose-containing solution to decrease their mobility in a controlled manner and mimic typical intracellular values). Time-lapse measurements were conducted on both systems at varying scan speeds and the RICS function was calculated and fitted. Exemplary liposome images and RICS functions for the two limit scan speeds (i.e., highest and lowest: 2 and 200 µs/pixel, respectively) are reported in Figure 1B. In keeping with expectations, the QDs characteristic diffusion coefficient within the liposome ($D_{QD}^{lip}$, µm^2^/s) (extracted by RICS analysis) vary according to the scan speed used (Figure 1D, full dots). In more detail, $D_{QD}^{lip}$ values extracted at high scan speeds (e.g., 2, 4 and 8 µs pixel dwell times) are almost coincident with the corresponding ones measured in solution ($D_{QD}^{sol}$, Figure 1D, empty dots). As an example, at a scan speed of 4 µs/pixel we obtained $D_{QD\;}^{lip}$ = 37.5 ± 7.1 µm^2^/s as compared to $D_{QD}^{sol}$ = 34.1 ± 7.8 µm^2^/s. As the scan speed decreases, instead, RICS spatial correlation starts to be affected by liposome motion, and $D_{QD}^{lip}$ values drop accordingly towards the diffusion coefficient of the liposome itself. The latter, as a reference, was extracted by exploiting the time resolution encrypted between consecutive frames (i.e., ~3.7 s) and applying Spatio-Temporal Image Correlation Spectroscopy (STICS [25] see Section 3) and resulted in $D_{lip}$ = (5.5 ± 2.7) × 10^−3^ µm^2^/s. It is worth mentioning that, as expected, $D_{QD}^{sol}$ values are almost invariant with respect to scan speed in the same range in which $D_{QD}^{lip}$ drops (Figure 1D, empty dots). As already mentioned, measurements on free Alexa488 in solution were also acquired as a control experiment, resulting in much higher diffusion coefficients (Figure S1). To further validate RICS at tunable timescales, the QD/liposome system was addressed independently by feedback-based 3D orbital tracking combined with RICS analysis along the orbit (schematics of the measurement and exemplary case are in Figure 1E–G). This approach allows the subtraction of the slow movement of a single liposome from the fast diffusion of molecules contained within it, the latter being captured directly by the orbiting laser spot and analyzed by RICS. In this case, the diffusivity of the single liposome can be extracted by standard MSD analysis of its trajectory (i.e., defined by the center of mass of the orbit in time, see exemplary case in Figure 1F). Nicely, the QD diffusivities extracted by RICS analysis on feedback-based tracking experiments at 2 µs/pixel scan speed (38.2 ± 3.9 µm^2^/s, Figure 1G, green dot) are in good agreement with the diffusivities extracted from RICS measurements at the same speed (43.8 ± 10.7 µm^2^/s, Figure 1G, red dot). It is worth meaning that the two techniques used are inherently different in the way they probe the complex intracellular environment, although they are both able to decouple the diffusion of molecules from the movement of subcellular nanostructures: RICS at tunable timescales, in fact, is performed on a selected 2D optical section of the cell (whose thickness is fixed and coincident with the resolution in the z-direction of the confocal imaging setup, i.e., ~1 µm) and relies on capturing a number of nanostructures in the chosen optical section; feedback-based orbital tracking, instead, affords sensitivity to the 3D movement of subcellular nanostructures by using two orbits (one slightly above and the other slightly below the target, see Section 3) but can follow a single nanostructure per time.

### 2.2. Fast RICS Captures Molecular Diffusion in Dynamic Nanostructures: Validation Tests in Living Cells

**Figure 2:**
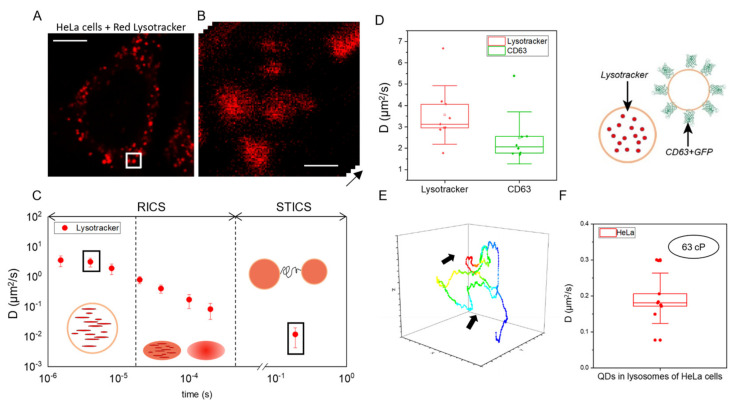
Time-tunable RICS: validation tests in living cells (**A**) Confocal image of a HeLa cell labeled with Red Lysotracker (scale bar 10 µm). (**B**) Magnification of the area used for the time-tunable RICS experiments (i.e., 33 nm per pixel, white square in (**A**). (**C**) Plot of the diffusion coefficients measured at different scan speeds for Lysotracker-labeled lysosomes. As schematically represented, at short pixel dwell times (i.e., few microseconds) RICS can probe the movement of the molecules inside the organelle. By contrast, at long pixel dwell times (i.e., hundreds of microseconds), RICS cannot distinguish fast molecular movements within the lysosome from the slow movement of the entire organelle; this latter component can be isolated and quantified by STICS. Standard deviations associated to data points are within 10–20% of the mean value: they are not discernible due to the logarithmic scale used on the *y*-axis (**D**) On the left: plot of the diffusion coefficients of Red Lysotracker (red) and GFP-labeled CD63 (green) measured on lysosomes of HeLa cells obtained at 4 µs of pixel dwell time. Upper and lower edges of the boxes represent the 25 and 75 percentiles of the distributions found, the middle line shows the mean value, whiskers show standard deviations; on the right: schematic representation of the two distinct situations (right side). (**E**) Example of trajectory of a QD-loaded lysosome in HeLa cell obtained using feedback-based 3D orbital tracking at 4 µs pixel dwell time. Ticks on axes represent pixels, thus 50 nm of space distance. Standard MSD analysis of this trajectory yields a diffusion coefficient of the tracked lysosome of 0.011 ± 0.009 µm^2^/s (expressed as Mean ± SD). (**F**) Plot of the diffusion coefficients (expressed as mean ± SD) of both QDs loaded into lysosomes measured using the feedback-based 3D orbital tracking routine at 4 µs pixel dwell time.

At this point, validation tests were performed in living cells. First, HeLa cells were treated with Red Lysotracker to stain lysosomes (Figure 2A,B). Then, following the established protocol, time-lapse imaging at different scan speeds was performed and the RICS/STICS functions were calculated. As expected, the analysis returned apparent diffusion coefficients for Red Lysotracker within lysosomes ($D_{Lys}^{cell}$) which vary according to the scan speed, as for the case of QD-loaded liposomes in solution (Figure 2C). Of particular interest, at the two fastest scan speeds, obtained at 2 and 4 µs/pixel, we obtained similar $D_{Lys}^{cell}$ values, indication that a limit diffusivity value for Red Lysotracker within lysosomes was reached. Thus, using the $D_{Lys}^{cell}$ value obtained at 2 µs/pixel (i.e., 3.6 ± 1.4 µm^2^/s) and the Stoke–Einstein relation (Equation (1)), an average lysosome viscosity of ~73 cP can be estimated (assuming a hydrodynamic radius of 0.8 nm for Red Lysotracker). This value is in good agreement with previous estimates obtained both by some of us using feedback-based orbital tracking on a similar probe (i.e., ACDAN) [26] and by others using Fluorescence Lifetime Imaging Microscopy (FLIM) on molecular motors [27]. To provide additional support to the reliability of this application, we performed a control experiment on lysosomes but using a different fluorescent probe: GFP-labeled CD63. CD63 is a protein that is expressed on the external surface of lysosomes. Figure 2D (left) shows the comparison between the diffusion coefficients of Red Lysotracker within lysosomes and CD63 on the external surface of lysosomes obtained at 2 µs/pixel dwell time. Lysotracker molecules inside the lysosome move faster than proteins on its external surface, the latter being slowed down by the intrinsic viscosity of the lipid bilayer (i.e., at 2 µs/pixel CD63-GFP yields *D* = 2.5 ± 1.2 µm^2^/s; schematic representation of protein localization in Figure 2D, right). Additional control experiments of comparison between CD63-GFP and DOPE (and CD63-GFP and GFP) are included in Figure S2. Finally, feedback-based 3D orbital tracking also provided confirmation of both the diffusivity of molecules inside lysosomes and the average diffusivity of the organelle itself. In more detail, QDs were administered to HeLa cells along with Lysotracker to verify their internalization inside lysosomes (see Section 3 and Figure S3) and used for feedback-based tracking because of their resistance to photo-bleaching. An exemplary trajectory of a lysosome loaded with QDs is reported in Figure 2E: standard MSD analysis of this trajectory yields a diffusion coefficient of the tracked lysosome of (1.1 ± 0.9) × 10^−2^ µm^2^/s, in line with what was already measured by some of us using iMSD [23]. From RICS analysis, instead, we retrieved the diffusion coefficient of QDs inside the organelle that correspond to an average viscosity value of ~63 cP (Figure 2F, obtained using Equation (1) with T = 37 ℃ and hydrodynamic radius for QDs of 7 nm, this latter estimated by performing RICS analysis of QDs freely dispersed in solution (see Section 3)). Thus, in spite of the very different hydrodynamic radii of the two probes used (0.8 nm for Lysotracker and 7 nm for QDs), the combination of RICS results and Stokes–Einstein relation (Equation (1)) returns a coherent picture of organelle viscosity.

### 2.3. Measuring Diffusion to Probe Viscosity of Intracellular Dynamic Nanostructures: Application to Lysosomes in Krabbe Disease

**Figure 3:**
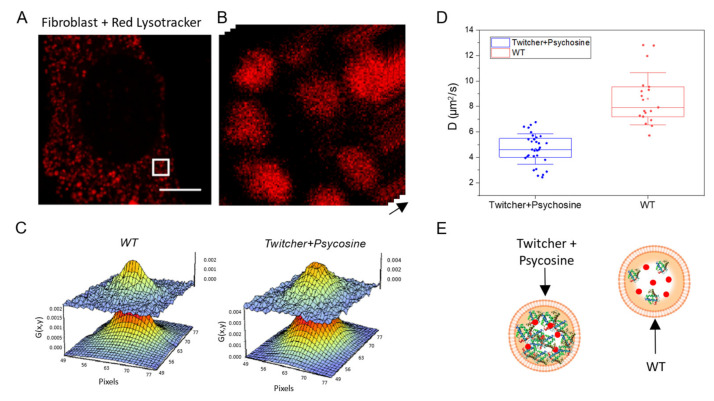
Time-tunable RICS application to the measurement of lysosome viscosity in KD model (**A**) Confocal image of a twitcher fibroblast cell labeled with Red Lysotracker (scale bar 10 µm). (**B**) Magnification of the white square in (**A**) used for the time-tunable RICS experiments (i.e., 33 nm per pixel). (**C**) Exemplary RICS correlation function obtained at 2 µs/pixel in WT fibroblasts (left) and twitcher fibroblasts treated with Psycosine. (**D**) Plot of the diffusion coefficients of Lysotracker in WT fibroblast (red) and in twitcher fibroblast treated with Psycosine at 2 µs/pixel. (**E**) Schematic diagram showing a possible molecular scenario explaining the intralysosomal diffusivity data obtained.

The validation tests demonstrate that, under selected experimental conditions, the diffusivity of molecules measured within a trafficking nanostructure can be used to estimate the viscosity of nanostructure itself (Equation (1)). This latter parameter can be of biomedical interest in several circumstances. As a case study, we propose here to monitor the viscosity of lysosomes in a murine cell model of Krabbe Disease (KD), or globoid cell leukodystrophy, a rare metabolic disorder in which the loss of function of the lysosomal hydrolase galactosylceramidase (GALC) leads to the accumulation of undigested material, primarily the cytotoxic psychosine (PSY), inside the organelles and the cell [28]. Such accumulation is supposed to affect, among other things, lysosome luminal crowding and viscosity. As described in detail in the Methods section, primary fibroblasts are extracted from ears of wild-type (WT) and twitcher (TWI) mice, this latter being a recognized animal model for the study of KD [29,30] (Figure 3A,B). WT fibroblasts are cultured under normal conditions, whereas TWI fibroblasts are exposed to 100 μM PSY for 24 h before imaging in order to exacerbate and better reproduce the KD phenotype. The latter treatment, indeed, reproduces in the fibroblast model the toxicity typically elicited by the abnormal accumulation of PSY in the glial cells of the nervous system. Thus, following the established protocol, time-lapse imaging at different scan speeds was performed and RICS/STICS functions were calculated (exemplary RICS functions for the two conditions tested are reported in Figure 3C). A fitting of the RICS autocorrelation curve obtained at the fastest scan speed (i.e., 2 µs) was used to estimate Lysotracker intralysosomal apparent diffusivity: we obtained *D* = 7.6 ± 3.1 μm^2^/s in WT cells and *D* = 5.2 ± 2.3 μm^2^/s in TWI cells treated with PSY (Figure 3D). These values, if combined with the apparent hydrodynamic radius of Lysotracker and Equation (1), correspond to a marked increase in lysosomal viscosity in TWI + PSY cells as compared to WT ones (~45%). This in turn provides confirmation, in live cells, that lysosome lumen is becoming more crowded due to the accumulation of undigested lipids in TWI cells as compared to control cells (schematic interpretation in Figure 3E), and well agrees with recent results by some of us obtained by feedback-based orbital tracking on the same biological system [26], and with results by others in a different LSD obtained by subcellular nanorheology [31].

### 2.4. Measuring Diffusion to Probe Molecular Aggregation within Subcellular Nanostructures: Application to the Insulin Secretory Granule

**Figure 4:**
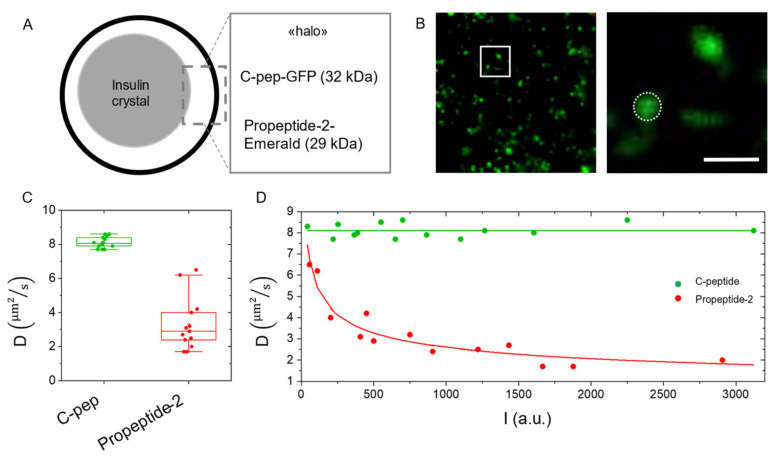
Time-tunable RICS application to the measurement of molecular aggregation within ISGs. (**A**) Schematic representation of an ISG, showing the typical structure with an internal core of crystallized insulin and a luminal “halo” surrounding the crystal (**B**) Confocal image of an insulinoma 1E cell labeled with proinsulin-GFP (left) and magnification of the white square in, i.e., the region used for the time-tunable RICS experiment (right, scale bar: 2 µm). (**C**) Plot of the diffusion coefficients of C-pep-GFP and propeptide2-Emerald (Mean ± SD) measured in INS-1E at 2 µs/pixel. (**D**) Here the measured diffusivity values are plotted against the mean fluorescence recorded in the subcellular structures captured by imaging. Propeptide-2-Emerald data fitting (reported in Figure 4) had been performed using Python, specifically the scipy.optimize.curve_fit () function which implements the Levenberg–Marquardt algorithm for nonlinear least squares fitting. The fitting equation has been chosen by taking into account the proportionality between diffusion coefficient (**D**) and molecular weight (MW) (Equations (2) and (3) in Section 3).

According to Equation (1), a factor affecting molecular diffusivity is the hydrodynamic radius (*r*) of the diffusing species. If the other relevant parameters, *T* and *ƞ*, are kept constant, in fact, a measurement of molecular diffusivity for a soluble protein can be revelatory of a change of *r* in turn due, for instance, to an ongoing process of aggregation (increase in *r*) or degradation (decrease in *r*). In both cases, the measured *r* (and, consequently, the measured *D*) is expected to vary according to the molecular weight (MW) of the newly formed species (aggregated or degraded) following the general relation: $r=\sqrt[{3}]{\alpha MW}$. In regards to the case of molecular aggregation, we propose here a study on two FP-tagged peptide markers of the insulin secretory granule (ISG) with similar MW transiently expressed in living INS-1E cells (an immortalized model of β-like cells), namely: proinsulin-GFP and proIAPP (Islet Amyloid Polypeptide)-Emerald (Figure 4). These two peptides, according to the known structural organization of the ISG [32], are supposed to be localized in the same subgranule compartment, i.e., the luminal ‘halo’ which surrounds a dense core of crystallized insulin (Figure 4A). Proinsulin-GFP is processed by intragranular enzymes with the release of a GFP-tagged C-peptide (MW: 32.4 kDa); similarly, proIAPP-Emerald undergoes endo-proteolytic processing leading to the generation of Emerald-tagged propetide-2 (MW: 29 kDa) [32]. INS-1E cells were transfected and imaged at 2 µs/pixel scan-speed to capture molecular diffusion (Figure 4B). Figure 4C reports the comparison between the diffusion coefficients obtained: it is clearly visible that data obtained for proIAPP-Emerald, as compared to those obtained for C-peptide-GFP, yield lower and much more dispersed (in terms of SD) diffusivity values. Of note, however, is that if the measured diffusivity values are put in relation with the level of expression of the recombinant constructs in cells, estimated by the intragranular fluorescence-intensity counts (see Section 3), a non-random distribution of diffusivity values emerges (Figure 4D); in fact, though the diffusivity of C-peptide-GFP is almost invariant with respect to its intragranular expression levels (Figure 4D, green), the diffusivity of propeptide-2-Emerald shows a substantial decrease with increasing intragranular expression levels of the construct. Interestingly, a proportionality between fluorescence intensity *I* and diffusion coefficient *D* is discernible ($D\propto I^{-1/3}$) in propeptide-2-Emerald data (see Equations (2) and (3) in Section 3). These data might be interpreted as the result of a concentration-dependent aggregation of the propeptide-2-Emerald protein but might also reveal only partial processing of the pro-IAPP-Emerald precursor with increasing intragranular concentration, then leading to concentration-dependent aggregation of the precursor itself.

## 3. Materials and Methods

### 3.1. Preparation of Fluorophore Solutions

Different fluorophore solutions were prepared and analyzed using RICS analysis as controls for the various experiments conducted. A 500 nM recombinant AcGFP (*Aequorea coerulescens* GFP) solution was prepared by dissolving the proper amount of protein in a 20 mM DEA buffer. AcGFP plasmid was purchased from Clonetech (Mountain View, CA, USA) and the protein was expressed in *E. coli* cells following standard procedures.

### 3.2. Preparation of Liposomes and Liposome-Based Samples

DPPC, 1,2-dipalmitoyl-sn-glycero-3-phosphocholine lipid (10 mg/mL in chloroform) was purchased from Avanti Polar Lipids (Alabaster, AL, USA). Low-gelling-temperature agarose, BioReagent was purchased from Sigma Aldrich (St. Louis, MO, USA). Liposomes of DPPC were prepared using the standard method as detailed in the following. Through the evaporation of 100 µL of chloroform solution containing 1 mg of DPPC in a centrifugal evaporator under vacuum for 2 h, a thin film of lipid was obtained. The film was then hydrated by adding 245 µL PBS at pH 7.45 and 5 µL of the stock solution of QDs at 50 °C. The final DPPC concentration was 5 mM. Liposomes were frozen in liquid nitrogen and then thawed in a water bath at 50 °C. This freeze–thaw cycle was repeated five times [33], then liposomes were partially immobilized using agarose gel [34]. Agarose was dissolved in PBS at a concentration of 1% *w*/*v*. Liposomes were added to the gel while the agarose was in the fluid state. After mixing, the solution was placed on a glass-bottom petri dish and was left at room temperature for jellification. Following jellification, liposomes containing QDs were analyzed using confocal microscopy. The fluorescence signal was collected and analyzed as described above. DOPC, 1,2-dioleoyl-sn-glycero-3-phosphocholine, DPPC, 1,2-dipalmitoyl-sn-glycero-3-phosphocholine (10 mg/mL in chloroform) and DOPE-Rhodamine, 1,2-dioleoyl-sn-glycero-3-phosphoethanolamine-N-(lissamine rhodamine B sulfonyl) (ammonium salt) (1 mg/mL in chloroform) were obtained from Avanti Polar Lipids (Alabaster, AL, USA). Low-gelling-temperature agarose, BioReagent, for molecular biology, was obtained from Sigma Aldrich (St. Louis, MO, USA). Qdot™ 545 ITK™ Amino (PEG) Quantum Dots were obtained from Molecular Probes (Eugene, OR, USA). Liposomes of DOPC with DOPE-Rhodamine (0.1 mol%) and DPPC with QDs were prepared by evaporating 100 µL of chloroform containing 1 mg of lipids in a centrifugal evaporator under the vacuum for 2 h. The remaining lipid film of DOPC/DOPE-Rhodamine was suspended in 250 µL of PBS (pH 7.45) at room temperature, whereas lipid film of DPPC was suspended in 245 µL of PBS (pH 7.45) and 5 µL of stock solution of QDs at 50 °C. The vesicles were subject to five cycles of freeze–thaw, frozen in liquid nitrogen and then thawed at 50 °C in a water bath. Liposomes were immobilized or slowed down using agarose gel. Agarose was dissolved in PBS (pH 7.45) at a concentration of 1.5% *w*/*v* and liposomes were added into the gel in different amounts while the gel was in fluid state. The solutions were placed on a glass-bottom petri dish and were left at room temperature for jellification.

### 3.3. Cell Culture and Treatments

HeLa cells (CCL-2 ATCC) were seeded on 22 mm glass-bottom dishes (Willco Wells) and let to adhere at 37 °C and 5% CO_2_ overnight. Dulbecco’s Modified Eagle Medium (DMEM) without phenol red (Gibco), supplemented with 10% Fetal Bovine Serum (FBS, Gibco), 100 U/mL of penicillin, and 100 μg/mL of streptomycin was used as a growth medium. For time-lapse acquisitions and diffusion coefficient analysis, HeLa cells were labeled using two different solutions: Lysotracker and CD63-protein transfection. LysoTracker Red DND-99 (Invitrogen) was added to the desired final concentration of 60 nM in the growth medium 20 min before the beginning of data acquisitions. CD63-pEGFP C2 plasmid (gift from Paul Luzio, Addgene plasmid # 62964) was transfected into cells by electroporation using a Neon Transfection System 10 μL Kit (Invitrogen). In particular, cells were trypsinized, pelleted and resuspended in Resuspension Buffer R. DNA (0.1 μg/μL) was added to 5 × 10^5^ cells in a 10 μL buffer, followed by electroporation using a Neon Transfection System (Invitrogen) operating at a voltage of 1005 V and width of 35 ms. After these steps, cells were seeded and cultured in DMEM containing 10% FBS and supplements without antibiotics and analyzed 24 h later. Lysotracker and QDs were administered to HeLa cells following 3 washing steps with PBS. For each 22 mm glass-bottom dish, 5 µL of QD stock solution (Qdot 545 ITK Amino (PEG), Thermofisher, Waltham, MA, USA) were suspended in 1 mL of growth medium without serum, and then, were poured on the glass-bottom dish containing cells following a sonication step of 5 min. After 3 h of incubation, cells were washed 3 times with PBS containing Mg^2+^ and Ca^2+^ (in order to avoid cell detachment) and then incubated again with a FBS-containing medium. After 24 h, cells were again washed three times with PBS containing Mg^2+^ and Ca^2^^+^ and then incubated with a medium containing FBS and Red Lysotracker at a concentration of 60 nM, as described above. 20 min later, cells were ready for microscope analysis. INS-1E cells [35] were cultured in a RPMI medium supplemented with 10% FBS, 10 mM HEPES, 10 mM glutamine, 100 U/mL of penicillin, 100 μg/mL of streptomycin, 10 mM sodium pyruvate and 50 µM β-mercaptoethanol at 37 °C and 5% CO_2_. Before imaging, INS-1E cells were seeded on an Ibi Treat µ-Dish 35 mm and let to adhere overnight. Proinsulin-EGFP [36] and proIAPP-Emerald [37] plasmids were transfected using lipofectamine 2000 following the manufacturer’s instructions. Cells were imaged 24 h post transfection. Primary wild-type (WT) and twitcher (TWI) fibroblast were extracted from WT and TWI ears. TWI heterozygous mice (TWI^+/−^ C57BL6 mice; Jackson Labs, Bar Harbor, ME, USA) were used as breeder pairs to generate homozygous twitcher mice (TWI^−/−^). Animals were maintained under standard housing conditions and used according to the protocols and ethical guidelines approved by the Ministry of Health (Permit Number: CBS-not. 0517; approved on the 4th of January 2018). The genetic status of each mouse was determined from the genome analysis of the twitcher mutation, as previously conducted by some of us [38]. Briefly, after anesthesia, mice ears were extracted, washed with sterile water and cut into small pieces. All pieces were than collected in an eppendorf tube and added with collagenase XI (C7657-100 mg; Sigma Aldrich) diluted 1:1 in a high glucose Dulbecco’s Modified Eagle Medium (DMEM) supplemented with 10% of heat-inactivated fetal calf serum (FCS), 2 mM L-glutamine and 1% penicillin/streptomycin (all products were from GIBCO-Life Technologies). After 2 h of incubation at 37 °C the eppendorf tube was centrifuged for 5 min at 200 g, the supernatant was discarded and the pellet was washed with 2 mL of PBS and centrifuged again discarding the supernatant. Trypsin-EDTA 0.05% (Thermo Fisher Scientific, Waltham, MA, USA) was then added to the tube and left for 45 min at 37 °C. The tube was than centrifuged and the pellet was re-suspended in the complete Dulbecco’s Modified Eagle Medium described above. Obtained cells were thus divided, pipetted up and down with a syringe, plated in a 60 mm cell plate (Falcon) and maintained at 37 °C in a humidified atmosphere containing 5% CO_2_. On the next day, the cells were washed and the media were replaced. After reaching confluence (approximately 3–4 days), cells were washed with 1 mL of PBS and splitted with a ratio of 1:2. For the imaging experiments, WT and TWI cells were plated in 12 mm Willco dishes and 6 h later TWI cells were administered with psychosine (PSY) 100 μM. PSY was dissolved in dimethylsulfoxide (DMSO) and control cultures received the same quantity of vehicle, which never exceeded 0.6% *v*/*v*. All imaging experiments were performed 24 h after plating.

### 3.4. Raster Image Correlation Spectroscopy in Tunable Timescales: Measurement and Data Analysis

For each experimental condition, images were acquired using an Olympus FluoView 1000-ASW-2.0 confocal microscope. To perform RICS analysis, for each case, temporal stacks of images were acquired at different pixel acquisition times (i.e., pixel dwell time): 2, 4, 8, 20, 40, 100 and 200 µs. As the pixel dwell time increases, the number of frames collected for that pixel time is decreased in order to obtain the same acquisition duration in each condition (and minimize bleaching). The scheme adopted was the following: 2 µs (200 frames acquired), 4 µs (150 frames), 8 µs (100 frames), 20 µs (80 frames), 40 µs (40 frames), 100 µs (20 frames) and 200 µs (10 frames). For RICS studies, a pixel size of 33 nm and 128 × 128 image size was adopted. Lysotracker and quantum dots (QD) 545 excitation was achieved using the 515 nm line of the Argon laser, whereas CD63-GFP, AcGFP proteins and Alexa 488 were excited with the 488 nm line. Fluorophore emission was collected by a 60× planApo water immersion objective (numerical aperture = 1.2). In detail, Lysotracker emission was collected in the 550–650 nm range, CD63-GFP, AcGFP and Alexa488 emission in the 500–600 nm range, QDs 545 in the 530–550 nm range and Rhodamine-DOPE in the 590–650 nm range.

The temporal stacks of images acquired for every experimental situation were first filtered using the moving average algorithm to subtract the contribution of the immobile fraction of molecules and/or slow movements of the vesicular structure itself. The moving average was kept to the minimum, i.e., it was applied among consecutive frames, to optimize correction of the nanostructure movement while preserving fast molecular dynamics. Once the filtering procedure was completed, image stacks were analyzed using the RICS algorithm, as described in the original works for RICS application to standard raster scans [18,19]. The analysis was performed using simFCS software v4.0 (www.lfd.uci.edu (accessed on 1 June 2022), University of California Irvine) that contained the above-described functionalities. This analysis returned the diffusion coefficients (D, µm^2^/s) of the motion of objects corresponding to each sampling speed, under the framework of a 3D diffusion model. The Stoke–Einstein equation (Equation (1)) was necessary to derive either the hydrodynamic radius or the solvent viscosity from diffusion measurements.

### 3.5. Spatio-Temporal Image Correlation Spectroscopy: Measurement and Data Analysis

The temporal stacks of images acquired for every experimental situation were analyzed using the STICS technique, as previously described [25]. The correlation curves derived from each experimental setup were calculated only for temporal stacks collected at 2 µs pixel dwell time (to minimize the time lag in between consecutive images). In more detail, we used temporal stacks acquired at a 2 µs pixel dwell time with 33 nm pixel size and 128 × 128 pixel resolution. This resulted in a time lag between two consecutive frames in a stack of 188 ms. Square brackets indicate the average operation over all spatial coordinates (i.e., *x* and *y*). For each image stack, a STICS curve was obtained. STICS curves were then fitted using simFCS software (www.lfd.uci.edu (accessed on 1 June 2022), University of California Irvine) that contains fitting tools described elsewhere [25]. This analysis returned the diffusion coefficients (D) of the motion of objects corresponding to a sampling period of 188 ms.

### 3.6. D Orbital Tracking Experiments

Lysosomes containing QDs 545 were individually tracked using the ISS Orbital Tracking System, analogous to that previously described [31] and embedded in an Olympus FluoView 1000-ASW-2.0 confocal laser microscope. To execute tracking, the ISS system sends two π/2-phase-shifted sine wave voltage signals to the scanning mirrors and makes the laser move along circular orbit; the offset values of the signals determine instead the center of the orbit. During tracking, the position of the center of the orbit is constantly updated at each tracking cycle (every 4 orbits in this case), following an algorithm based on the fast Fourier transformation (FFT) of the collected signal [22]. From the FFT of the QDs 545 fluorescence signal, the continuous (or ‘DC’) component (i.e., the zero th term of the Fourier series) and its ‘AC’ component, as the first harmonic term of the Fourier series, can be derived. The ‘DC’ and ‘AC’ components allow the determination of the distance of the tracked object from the center of the orbit using the modulation of the signal (defined as mod = ac/dc) and the angular component by the phase of the ac term. To correct the orbit, the tracking system changes the sine wave signals to keep the modulation at its minimum; this would result in having the object at the center of the orbit. QDs 545 fluorescent emission was collected on 256 pixels around a 150 nm-radius orbit with a period of 1024 ms (meaning a pixel dwell time of 4 µs derived from: 1024 ms/256 pixels = 4 µs/pixel) and the orbit position was updated every 4-orbit period (defined as a ‘cycle’, approximately 4 ms). The movement along the vertical axis is instead controlled with a piezoelectric stage that receives a square wave voltage signal that periodically raises and lowers the stage and thus the tracking laser orbit that periodically will be located above and below the tracked object. The vertical distance between the two orbits can be controlled by the amplitude of the voltage signal. The FFT algorithm described above, using the same mechanism, is able to recover the vertical position of the object in order to keep it at the center of the upper and lower laser orbits. The ISS Orbital Tracking System is able to record every single correction of the tracking orbit (and thus every single movement of the object) and reconstruct its trajectory from which many other important parameters can be recovered, such as the mean square displacement (MSD) or the average apparent diffusion coefficient D. The intensity fluorescence signal collected with orbital tracking measurements was stored in intensity carpets where each row reports each orbit of the measurement and each column represents a pixel along the orbit, as previously described [28]. Adjacent rows represent consecutive orbits and adjacent columns correspond to adjacent pixels along the collected orbits. Spatial correlation was computed on intensity carpets and then fitted as described in the original work for RICS applied to circular raster scans [39].

### 3.7. Calibration of Hydrodynamic Radius of QDs in Buffer Solution

To calibrate the hydrodynamic radius of QDs, we suspended 5 µL of QD stock solution in 250 µL of 50 mM Borate buffer. The suspension was placed on a 22 mm glass-bottom dish for cell culture and OT measurements were acquired on that. The intensity signal was analyzed as described above. Using Equation (1), the average hydrodynamic radius was extrapolated knowing the viscosity of the buffer and the temperature at which the experiment was conducted. The result was then used to compute the viscosity of lysosomes when analyzed with QDs.

### 3.8. Statistical Analysis

Diffusion coefficients from each experimental situation were tested with a D’Agostino-Pearson test to check their normal distribution. The tests gave a positive result for each case. Thus, diffusion coefficient values are reported throughout the text as Mean ± SD, and compared among different experiments using the Student’s *t*-test. Each analysis was conducted using OriginPro^®^9. Differences were considered significant for *p* < 0.05. Propeptide-2 data fitting (reported in Figure 4) was performed using Python, specifically the scipy.optimize.curve_fit() method which implements the Levenberg-Marquardt algorithm for nonlinear least squares fitting. The fitting equation was chosen by taking into account the proportionality between diffusion coefficient (*D*) and molecular weight (*MW*):

(2) $$D\propto MW^{-1/3}$$

And the equation was:

(3) $$D=A\cdot I^{-1/3}$$

where ‘*I*’ is fluorescence intensity and ‘*A*’ is a free parameter. The results gave *A* = 26.06 and covariance = 0.8, thus there was good correspondence between experimental data and the fitting equation. Concerning the average fluorescence intensity analysis, it was calculated on the first frame of each acquired movie, by means of ImageJ plugin Analyze Particles, used to isolate ISGs fluorescence spots. The obtained value was normalized based on the laser intensity and PMT gain of each acquisition using green Autofluorescent Plastic Slides from CHROMA^®^ as a reference. For propeptide-2-Emerald fluorescence intensity analysis, fluorescence counts were firstly normalized to EGFP counts using a brightness ratio between the two FPs [24].

## 4. Conclusions

Here we offer to experimental biologists (and biophysicists) a simple tool to analyze molecular diffusion in nanoscopic dynamic compartments of living cells. Starting from a standard stack of images, RICS at fast timescales offers access to the diffusion coefficient of a properly labeled molecule contained within a dynamic subcellular nanosystem. Being a fluctuation-based approach, it does not rely on localization and, as a consequence, on the extraction of trajectories, such as in a standard SPT experiment. By contrast, the protocol proposed provides rapid and robust quantification of the diffusion of molecules over the entire probed area, which typically encloses few target nanostructures. The inherent simplicity and modest dependence on labeling strategies (and on S/N ratio) of the proposed approach paves the way to applications in biology and related fields, particularly if large-scale screening is needed (e.g., different phenotypes, different pharmacological treatments, etc.). Here, two applications of biomedical interest are reported, in which measuring diffusion is a means to recognize and quantitatively describe average alterations in the molecular properties of a particular subcellular structure of interest. 

From a technological point of view, being based on standard imaging, the protocol described is fully compatible with any kind of scanning-based setup (e.g., confocal, STED-based), either with one- or multi-photon excitation, the only requisite being that the time resolution of the measurement can be properly tuned to the characteristic dynamics under study. Lastly, the approach can be combined with many other tools, either fluctuation-based (e.g., STICS, PLICS [40], Number&Brightness analysis [41]) to increase the amount of information that can be extracted and complement selected limitations, or linked to the use of ‘intelligent’ dyes to probe selected parameters (e.g., pH, membrane order, etc.). In addition, as with any other fluctuation-based approach, it can be easily adapted to a multi-channel acquisition mode (i.e., using multiple colors) to extend the utility of the technique to the measurement of biomolecular interactions and kinetics. Collectively, such implementations could transform the basic idea presented here into a flexible, multiplexed platform to address quantitatively the complex regulation of life at the subcellular level.

## Supplementary Materials

The following supporting information can be downloaded at: https://www.mdpi.com/article/10.3390/ijms23147623/s1.
