# Supplementary material for: Measuring Molecular Diffusion in Dynamic Subcellular Nanostructures by Fast Raster Image Correlation Spectroscopy and 3D Orbital Tracking
Source: Int J Mol Sci. 2022 Jul 10;23(14):7623. doi: 10.3390/ijms23147623 (PMC9323805; doi:10.3390/ijms23147623)
Supplement: Supplementary file 1 [file ijms-23-07623-s001.zip › ijms-1805097-supplementary.pdf]

Supplementary Figures

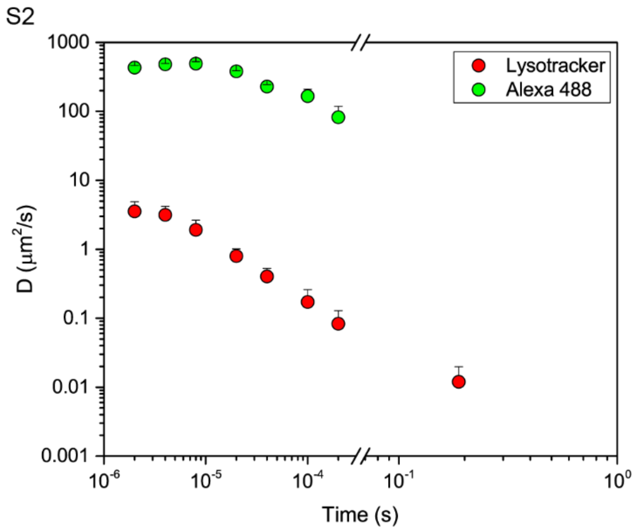

Figure S1. Comparison between diffusion coefficients of free Alexa 488 dissolved in water and of Red Lysotracker in lysosomes of HeLa cells. This graph shows the diffusion coefficients found for both Alexa 488 dissolved in water (green dots) and Red Lysotracker within the lysosomes of HeLa cells (red dots) at different scan speeds. Note how Alexa 488 returns diffusion coefficients that are two order of magnitude higher than Lysotracker, although they are small molecules with comparable hydrodynamic radii. Worth mentioning: for pixel-dwell-times higher than 20 µs the diffusion coefficient measured for Alexa 488 starts decreasing due to down-sampling, i.e. due to high diffusivity of Alexa 488, it is not possible to grab its rapid movements at scan speed slower than a certain threshold.

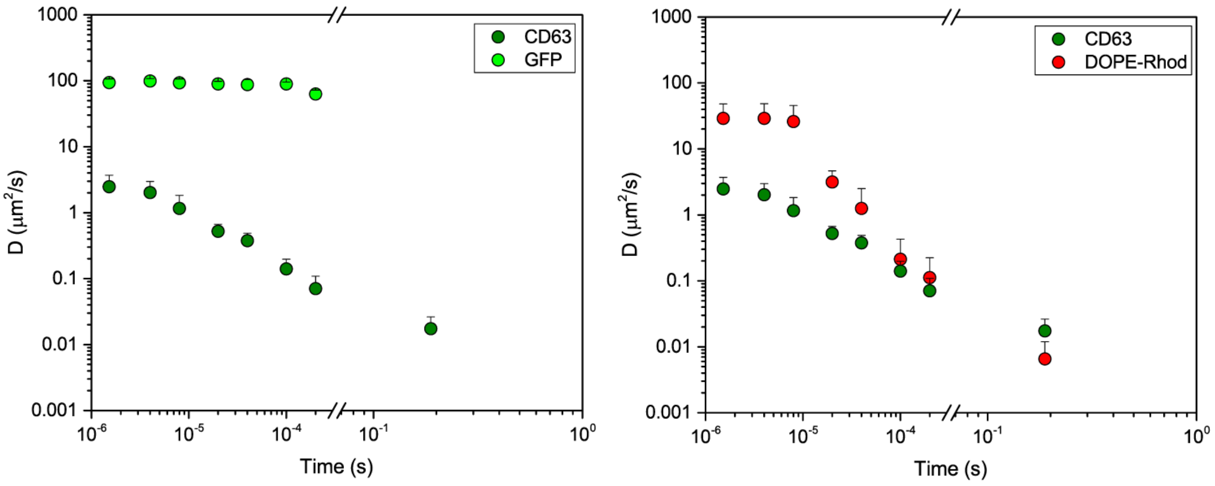

(**A**) (**B**)

Figure S2. RICS at tunable time scales to probe molecular diffusivity on the surface of dynamic subcellular nanostructures. (**A**) Plot of the diffusion coefficients found for both free GFP dissolved in water (light-green dots) and GFP-labeled CD63, a transmembrane protein marker of lysosomes expressed in HeLa cells (dark-green dots) at different scan speeds. Note that free GFP in solution yields the expected D value at 37ºC in solution, that is of about 100 $\mu m^2/s$. (**B**) Here the diffusion coefficients found for GFP-labeled CD63 in HeLa cells (dark-green dots, the same as in (A)) are compared with those found for Rhodamine-labeled DOPE embedded into the membrane of liposomes (red dots). The analysis returned that Rhodamine-labeled DOPE diffuses on the liposome surface much faster than GFP-labeled CD63, as somewhat expected for a lipid as compared to a transmembrane protein.

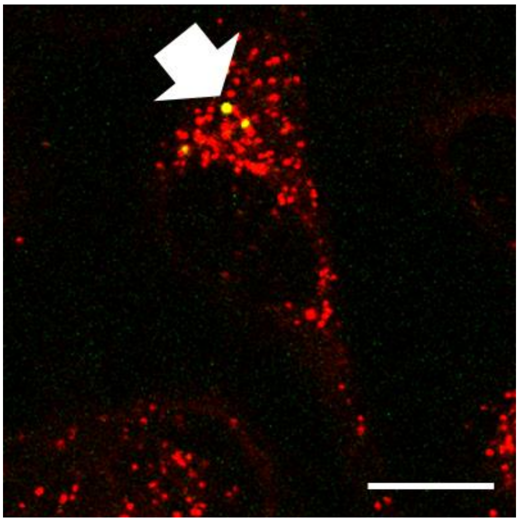

Figure S3. HeLa cells treated with QDs for 3 hours and labelled with Red Lysotracker. The white arrow indicates one of the vesicles which contain both QDs and Lysotracker. Scale bar: 10 µm.
